# Supplementary material for: Differential effects of habitat loss on occupancy patterns of the eastern green lizard Lacerta viridis at the core and periphery of its distribution range
Source: PLoS One. 2020 Mar 5;15(3):e0229600. doi: 10.1371/journal.pone.0229600 (PMC7058328; doi:10.1371/journal.pone.0229600)

S1 Appendix 1. Distribution of variables representative of habitat configuration in each region.

**Periphery**

**Core**


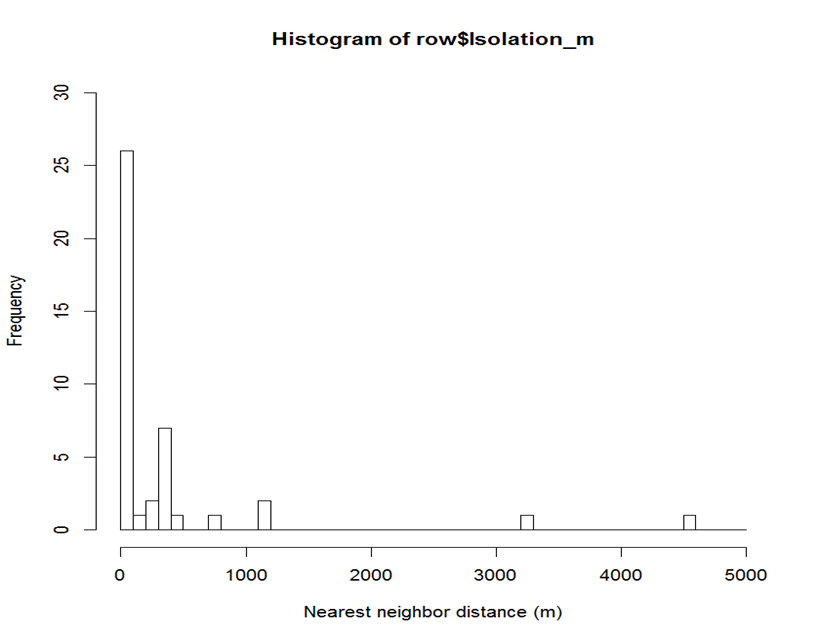

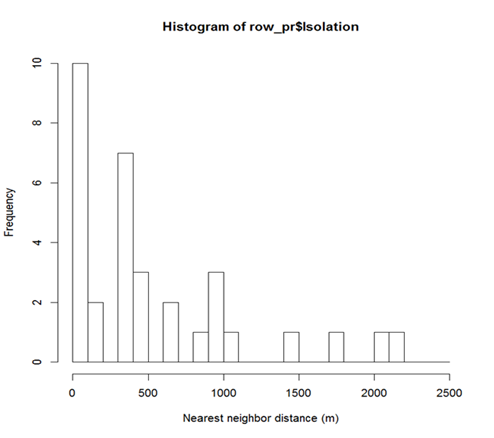


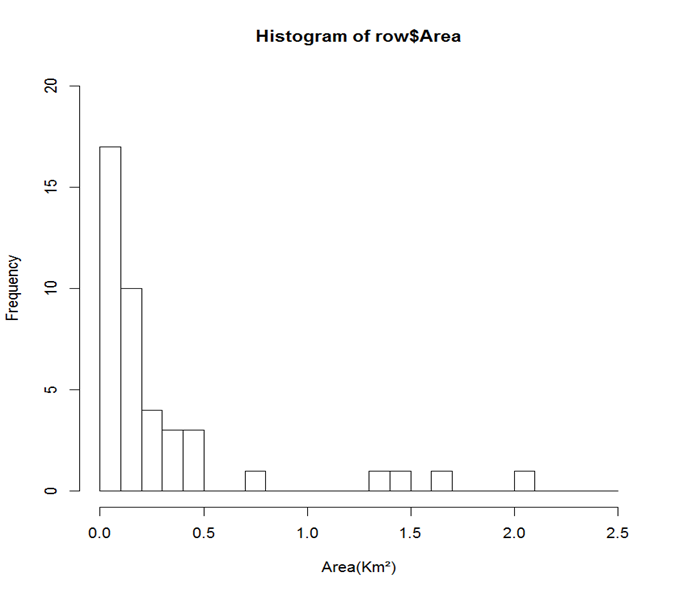

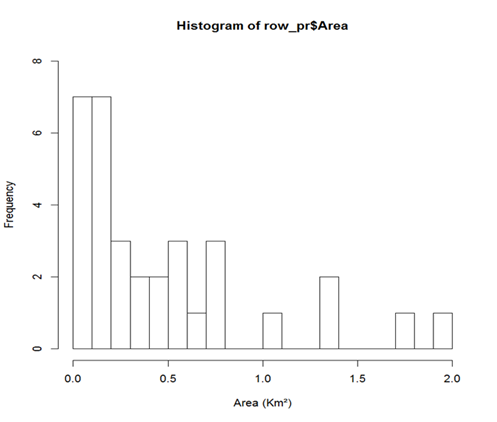


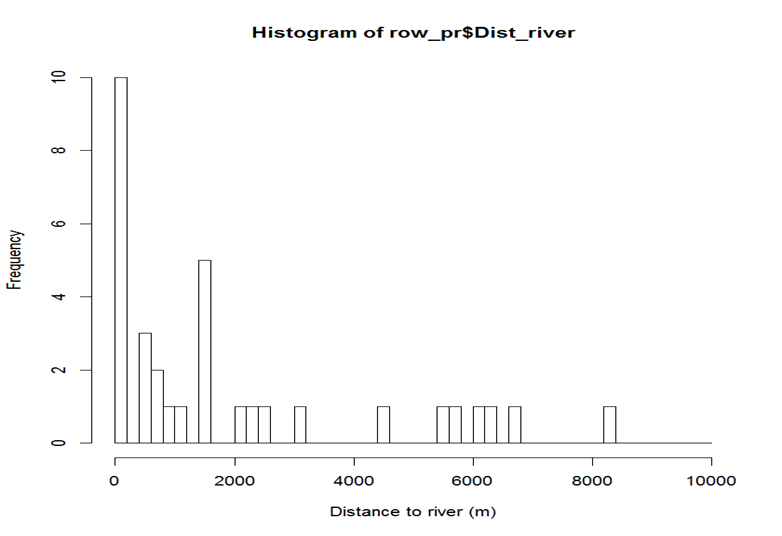

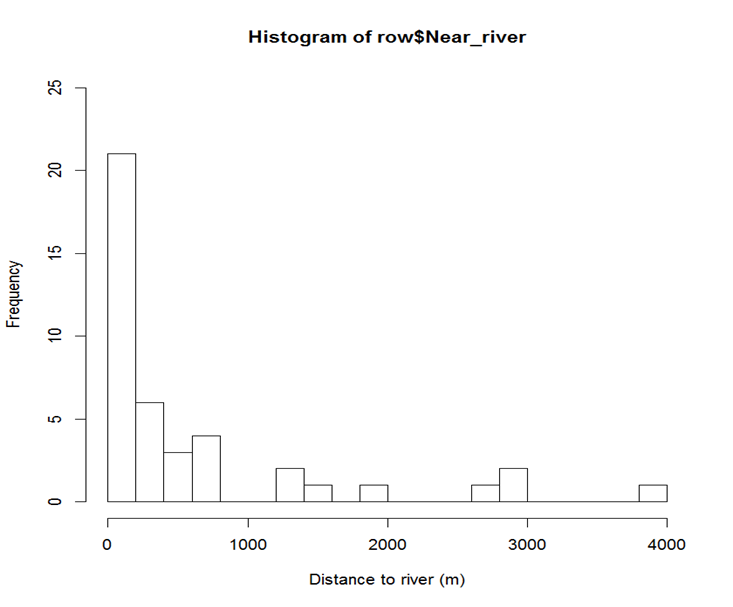

Supplement: S1 Appendix — (DOCX) [file pone.0229600.s001.docx]
