# Supplementary material for: Differential effects of habitat loss on occupancy patterns of the eastern green lizard Lacerta viridis at the core and periphery of its distribution range
Source: PLoS One. 2020 Mar 5;15(3):e0229600. doi: 10.1371/journal.pone.0229600 (PMC7058328; doi:10.1371/journal.pone.0229600)
Supplement: S2 Appendix — (DOCX) [file pone.0229600.s002.docx]

S1 Appendix 2 Location of habitat patches surveyed in each region

Core:

| Patch | latitud | longitud |
| --- | --- | --- |
| 1 | 42.1543 | 24.7331 |
| 2 | 42.1447 | 24.7384 |
| 3 | 42.1367 | 24.7307 |
| 4 | 42.1424 | 24.7005 |
| 5 | 42.1558 | 24.7519 |
| 6 | 42.1530 | 24.7341 |
| 7 | 42.1457 | 24.7467 |
| 8 | 42.1639 | 24.7627 |
| 9 | 42.1616 | 24.7702 |
| 10 | 42.1612 | 24.7716 |
| 11 | 42.1597 | 24.7760 |
| 12 | 42.1622 | 24.7972 |
| 13 | 42.1560 | 24.7639 |
| 14 | 42.1486 | 24.7074 |
| 15 | 42.1529 | 24.7065 |
| 16 | 42.1585 | 24.7222 |
| 17 | 42.1587 | 24.7194 |
| 18 | 42.1573 | 24.7179 |
| 19 | 42.1611 | 24.7159 |
| 20 | 42.1684 | 24.7155 |
| 21 | 42.1762 | 24.7112 |
| 22 | 42.1813 | 24.7156 |
| 23 | 42.1641 | 24.7708 |
| 24 | 42.1904 | 24.7691 |
| 25 | 42.1951 | 24.7754 |
| 26 | 42.1986 | 24.7590 |
| 27 | 42.2315 | 24.7751 |
| 28 | 42.2190 | 24.7853 |
| 29 | 42.1248 | 24.8670 |
| 30 | 42.1510 | 24.8828 |
| 31 | 42.1520 | 24.8169 |
| 32 | 42.1246 | 24.8686 |
| 33 | 42.1936 | 24.8213 |
| 34 | 42.2123 | 24.8676 |
| 35 | 42.2286 | 24.8579 |
| 36 | 42.2246 | 24.8831 |
| 37 | 42.2060 | 24.8987 |
| 38 | 42.1997 | 24.8894 |
| 39 | 42.2262 | 24.8482 |
| 40 | 42.1984 | 24.8883 |
| 41 | 42.2119 | 24.8665 |
| 42 | 42.2387 | 24.7159 |

Periphery:

| Patch | Latitud | Longitud |
| --- | --- | --- |
| 1 | 50.1324 | 14.4002 |
| 2 | 50.1372 | 14.403 |
| 3 | 50.1179 | 14.4023 |
| 4 | 50.1469 | 14.3794 |
| 5 | 49.9542 | 14.4185 |
| 6 | 49.9616 | 14.414 |
| 7 | 50.0178 | 14.4141 |
| 8 | 50.0004 | 14.3784 |
| 9 | 50.0141 | 14.3857 |
| 10 | 50.0104 | 14.3727 |
| 11 | 49.9439 | 14.4111 |
| 12 | 50.0909 | 14.3421 |
| 13 | 50.0835 | 14.3537 |
| 14 | 50.0713 | 14.3284 |
| 15 | 50.0716 | 14.3657 |
| 16 | 50.059 | 14.3456 |
| 17 | 50.0656 | 14.3339 |
| 18 | 50.0657 | 14.3806 |
| 19 | 50.0596 | 14.3901 |
| 20 | 50.0623 | 14.399 |
| 21 | 49.9877 | 14.3565 |
| 22 | 49.9857 | 14.38 |
| 23 | 49.9843 | 14.3726 |
| 24 | 49.9782 | 14.4013 |
| 25 | 49.9574 | 14.4019 |
| 26 | 50.0313 | 14.3253 |
| 27 | 50.0408 | 14.371 |
| 28 | 50.0452 | 14.3938 |
| 29 | 50.0533 | 14.3861 |
| 30 | 50.0404 | 14.3997 |
| 31 | 49.9643 | 14.42 |
| 32 | 49.9553 | 14.3865 |
| 33 | 50.1452 | 14.4014 |
