# Supplementary figures and images for: Differential effects of habitat loss on occupancy patterns of the eastern green lizard Lacerta viridis at the core and periphery of its distribution range
Source: PLoS One. 2020 Mar 5;15(3):e0229600. doi: 10.1371/journal.pone.0229600 (PMC7058328; doi:10.1371/journal.pone.0229600)

S1 Appendix 3. Maps of classified land cover classes in each region.


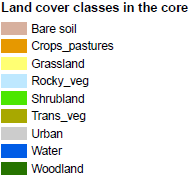

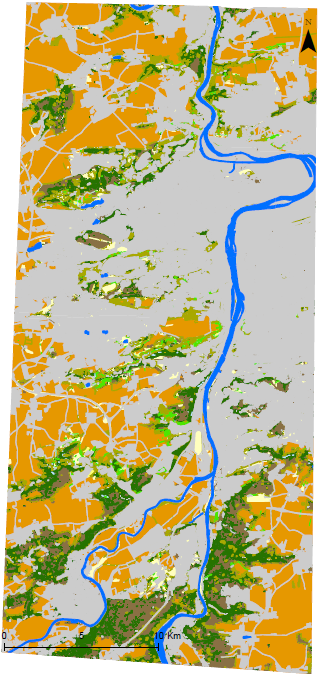

Supplement: S3 Appendix — (DOCX) [file pone.0229600.s003.docx]
