# Supplementary material for: Differential effects of habitat loss on occupancy patterns of the eastern green lizard Lacerta viridis at the core and periphery of its distribution range
Source: PLoS One. 2020 Mar 5;15(3):e0229600. doi: 10.1371/journal.pone.0229600 (PMC7058328; doi:10.1371/journal.pone.0229600)
Supplement: S4 Appendix — Variables were included to model only detection probability (p) while maintaining occupancy probability (psi) constant. (DOCX) [file pone.0229600.s004.docx]

S1 Appendix 4. Models for detection probability in each region. Variables were included to model only detection probability (p) while maintaining occupancy probability (psi) constant.

| Model |  | AICc | ΔAIC | weight |
| --- | --- | --- | --- | --- |
|  |  |  |  |  |
| Core |  |  |  |  |
|  |  |  |  |  |
| p(day+veg_str+area), psi(.) |  | 96.72 | 0 | 0.244 |
| p(day+veg_str), psi(.) |  | 96.99 | 0.27 | 0.213 |
| p(area), psi(.) |  | 97.6 | 0.87 | 0.157 |
| p(day), psi(.) |  | 98.08 | 1.35 | 0.124 |
| p(veg_str+area), psi(.) |  | 98.43 | 1.71 | 0.104 |
| p(day+area), psi(.) |  | 98.63 | 1.9 | 0.094 |
|  |  |  |  |  |
|  |  |  |  |  |
| Periphery |  |  |  |  |
|  |  |  |  |  |
| p(area+veg_str), psi(.) |  | 49.15 | 0.00 | 0.28 |
| p(day+area+veg_str), psi(.) |  | 49.53 | 0.38 | 0.23 |
| p(.), psi(.) |  | 49.65 | 0.50 | 0.22 |
| p(day+veg_str), psi(.) |  | 50.52 | 1.37 | 0.14 |
| p(veg_str), psi(.) |  | 50.53 | 1.37 | 0.14 |
