# Supplementary material for: Differential effects of habitat loss on occupancy patterns of the eastern green lizard Lacerta viridis at the core and periphery of its distribution range
Source: PLoS One. 2020 Mar 5;15(3):e0229600. doi: 10.1371/journal.pone.0229600 (PMC7058328; doi:10.1371/journal.pone.0229600)
Supplement: S5 Appendix — (DOCX) [file pone.0229600.s005.docx]

S1 Appendix 5. Spearman rank correlations of no scale dependent variables in each region.

| **Core** |  |  |  |  |  |  |  |  |  |  |  |  |  |  |  |  |
| --- | --- | --- | --- | --- | --- | --- | --- | --- | --- | --- | --- | --- | --- | --- | --- | --- |
|  |  |  |  |  |  |  |  |  |  |  |  |  |  |  |  |  |
|  | Area | Perimeter | Per_Area | Shape_index | CAI_5m | CAI_10m | CAI_25m | CAI_51m | CAI_75m | CAI_100m | Veg_str | Radiation | Np_dist | dist_river |  |  |
| Area | 1 | 0.361 | -0.839 | 0.442 | 0.676 | 0.660 | 0.681 | 0.755 | 0.763 | 0.692 | 0.186 | 0.333 | -0.103 | -0.087 |  |  |
| Perimeter | 0.361 | 1 | 0.038 | 0.249 | 0.202 | 0.196 | 0.213 | 0.263 | 0.270 | 0.175 | -0.095 | 0.233 | 0.071 | 0.138 |  |  |
| Per_Area | -0.839 | 0.038 | 1 | -0.091 | -0.814 | -0.798 | -0.794 | -0.798 | -0.762 | -0.699 | -0.418 | -0.364 | 0.009 | -0.053 |  |  |
| Shape_index | 0.442 | 0.249 | -0.091 | 1 | -0.312 | -0.331 | -0.280 | -0.126 | -0.072 | -0.095 | -0.477 | -0.139 | -0.353 | -0.573 |  |  |
| CAI_5m | 0.676 | 0.202 | -0.814 | -0.312 | 1 | 0.995 | 0.961 | 0.897 | 0.853 | 0.802 | 0.573 | 0.508 | 0.184 | 0.348 |  |  |
| CAI_10m | 0.660 | 0.196 | -0.798 | -0.331 | 0.995 | 1 | 0.973 | 0.909 | 0.863 | 0.802 | 0.582 | 0.513 | 0.191 | 0.354 |  |  |
| CAI_25m | 0.681 | 0.213 | -0.794 | -0.280 | 0.961 | 0.973 | 1 | 0.955 | 0.911 | 0.817 | 0.550 | 0.452 | 0.206 | 0.344 |  |  |
| CAI_51m | 0.755 | 0.263 | -0.798 | -0.126 | 0.897 | 0.909 | 0.955 | 1 | 0.955 | 0.845 | 0.441 | 0.404 | 0.203 | 0.283 |  |  |
| CAI_75m | 0.763 | 0.270 | -0.762 | -0.072 | 0.853 | 0.863 | 0.911 | 0.955 | 1 | 0.892 | 0.347 | 0.327 | 0.197 | 0.229 |  |  |
| CAI_100m | 0.692 | 0.175 | -0.699 | -0.095 | 0.802 | 0.802 | 0.817 | 0.845 | 0.892 | 1 | 0.307 | 0.284 | 0.142 | 0.138 |  |  |
| Veg_str | 0.186 | -0.095 | -0.418 | -0.477 | 0.573 | 0.582 | 0.550 | 0.441 | 0.347 | 0.307 | 1 | 0.498 | 0.175 | 0.333 |  |  |
| Radiation | 0.333 | 0.233 | -0.364 | -0.139 | 0.508 | 0.513 | 0.452 | 0.404 | 0.327 | 0.284 | 0.498 | 1 | 0.410 | 0.283 |  |  |
| np_dist | -0.103 | 0.071 | 0.009 | -0.353 | 0.184 | 0.191 | 0.206 | 0.203 | 0.197 | 0.142 | 0.175 | 0.410 | 1 | 0.415 |  |  |
| dist_river | -0.087 | 0.138 | -0.053 | -0.573 | 0.348 | 0.354 | 0.344 | 0.283 | 0.229 | 0.138 | 0.333 | 0.283 | 0.415 | 1 |  |  |
|  |  |  |  |  |  |  |  |  |  |  |  |  |  |  |  |  |
|  |  |  |  |  |  |  |  |  |  |  |  |  |  |  |  |  |
| **Periphery** |  |  |  |  |  |  |  |  |  |  |  |  |  |  |  |  |
|  |  |  |  |  |  |  |  |  |  |  |  |  |  |  |  |  |
|  | Area | Perimeter | Per_area | Shape_index | CAI_5m | CAI_10m | CAI_25m | CAI_50m | CAI_75m | CAI_100m | Veg_str | Radiation | Slope | Np_dist | dist_river | dist_crop |
| Area | 1 | 0.039 | -0.892 | 0.552 | 0.064 | 0.067 | 0.091 | 0.046 | 0.031 | -0.040 | 0.186 | 0.058 | 0.223 | -0.400 | -0.032 | -0.221 |
| Perimeter | 0.039 | 1 | 0.290 | -0.028 | 0.037 | 0.040 | 0.067 | 0.013 | 0.061 | 0.096 | 0.175 | 0.151 | 0.103 | -0.094 | -0.235 | -0.094 |
| Per_area | -0.892 | 0.290 | 1 | -0.332 | 0.031 | 0.027 | 0.015 | 0.064 | 0.111 | 0.202 | -0.104 | 0.041 | -0.237 | 0.317 | 0.023 | 0.192 |
| Shape_index | 0.552 | -0.028 | -0.332 | 1 | 0.252 | 0.252 | 0.280 | 0.323 | 0.327 | 0.302 | 0.245 | 0.037 | -0.021 | -0.358 | 0.185 | -0.257 |
| CAI_5m | 0.064 | 0.037 | 0.031 | 0.252 | 1 | 0.999 | 0.979 | 0.902 | 0.859 | 0.796 | -0.220 | 0.111 | -0.161 | -0.106 | 0.072 | -0.225 |
| CAI_10m | 0.067 | 0.040 | 0.027 | 0.252 | 0.999 | 1 | 0.982 | 0.906 | 0.864 | 0.800 | -0.206 | 0.108 | -0.157 | -0.112 | 0.071 | -0.220 |
| CAI_25m | 0.091 | 0.067 | 0.015 | 0.280 | 0.979 | 0.982 | 1 | 0.958 | 0.922 | 0.861 | -0.132 | 0.130 | -0.104 | -0.165 | 0.048 | -0.189 |
| CAI_50m | 0.046 | 0.013 | 0.064 | 0.323 | 0.902 | 0.906 | 0.958 | 1 | 0.985 | 0.936 | -0.086 | 0.173 | -0.103 | -0.221 | 0.052 | -0.129 |
| CAI_75m | 0.031 | 0.061 | 0.111 | 0.327 | 0.859 | 0.864 | 0.922 | 0.985 | 1 | 0.966 | -0.066 | 0.206 | -0.095 | -0.249 | 0.032 | -0.069 |
| CAI_100m | -0.040 | 0.096 | 0.202 | 0.302 | 0.796 | 0.800 | 0.861 | 0.936 | 0.966 | 1 | -0.068 | 0.249 | -0.071 | -0.180 | 0.044 | 0.022 |
| Veg_str | 0.186 | 0.175 | -0.104 | 0.245 | -0.220 | -0.206 | -0.132 | -0.086 | -0.066 | -0.068 | 1 | -0.048 | 0.311 | -0.100 | -0.085 | 0.075 |
| Radiation | 0.058 | 0.151 | 0.041 | 0.037 | 0.111 | 0.108 | 0.130 | 0.173 | 0.206 | 0.249 | -0.048 | 1 | -0.265 | -0.056 | -0.182 | 0.137 |
| Slope | 0.223 | 0.103 | -0.237 | -0.021 | -0.161 | -0.157 | -0.104 | -0.103 | -0.095 | -0.071 | 0.311 | -0.265 | 1 | -0.176 | -0.235 | 0.055 |
| np_dist | -0.400 | -0.094 | 0.317 | -0.358 | -0.106 | -0.112 | -0.165 | -0.221 | -0.249 | -0.180 | -0.100 | -0.056 | -0.176 | 1 | 0.300 | 0.171 |
| dist_river | -0.032 | -0.235 | 0.023 | 0.185 | 0.072 | 0.071 | 0.048 | 0.052 | 0.032 | 0.044 | -0.085 | -0.182 | -0.235 | 0.300 | 1 | 0.457 |
| dist_crop | -0.221 | -0.094 | 0.192 | -0.257 | -0.225 | -0.220 | -0.189 | -0.129 | -0.069 | 0.022 | 0.075 | 0.137 | 0.055 | 0.171 | 0.457 | 1 |
