# Supplementary material for: Differential effects of habitat loss on occupancy patterns of the eastern green lizard Lacerta viridis at the core and periphery of its distribution range
Source: PLoS One. 2020 Mar 5;15(3):e0229600. doi: 10.1371/journal.pone.0229600 (PMC7058328; doi:10.1371/journal.pone.0229600)
Supplement: S6 Appendix — Each set includes all variables plus the variable or combination of variables indicated. (DOCX) [file pone.0229600.s006.docx]

S1 Appendix 6. Different sets of models ran in each single scale and multiscale models in each region. Each set includes all variables plus the variable or combination of variables indicated.

**Core**

Single-scale

models

Multi-scale

model

Set 1: Np_dist, crop_pas

Set 2: Np_dist, urban

Set 3: Prox, crop_pas

Set 4: Prox, urban

Set 1: Np_dist

Set 2: Prox

**Periphery**

Single-scale

models

Multi-scale

model

Set 1: Habitat

Set 2:Urban

Set 1: Habitat

Set 2: Urban
