# Supplementary material for: Differential effects of habitat loss on occupancy patterns of the eastern green lizard Lacerta viridis at the core and periphery of its distribution range
Source: PLoS One. 2020 Mar 5;15(3):e0229600. doi: 10.1371/journal.pone.0229600 (PMC7058328; doi:10.1371/journal.pone.0229600)
Supplement: S7 Appendix — (DOCX) [file pone.0229600.s007.docx]

S1 Appendix 7. Best selected models at small scales from 50m to 250m in the core region.

| **Scale** | **RN²** | **PCC** | **AUC** | **Kappa0.5** | **Kappaopt** | **Dist_river** | **Np_dist** | **Prox** | **Habitat** | **Crop_pas** | **Urban** | **Area** | **Perimeter** | **Shape_index** | **Veg_str** | **Radiation** |
| --- | --- | --- | --- | --- | --- | --- | --- | --- | --- | --- | --- | --- | --- | --- | --- | --- |
|  |  |  |  |  |  |  |  |  |  |  |  |  |  |  |  |  |
| 50 | 0.7 | 0.88 | 0.872 | 0.751 | 0.8 | X |  | X |  | X |  |  |  | X | X | X |
|  | 0.69 | 0.904 | 0.878 | 0.8 | 0.8 | X |  | X | X |  |  |  |  | X | X | X |
|  | 0.66 | 0.857 | 0.907 | 0.704 | 0.755 | X |  | X |  |  |  |  |  |  | X | X |
| 150 | 0.55 | 0.833 | 0.74 | 0.642 | 0.642 |  |  |  | X |  |  |  |  |  |  |  |
| 250 | 0.64 | 0.857 | 0.75 | 0.695 | 0.696 |  | X |  | X | X |  | X |  | X | X |  |
