# Supplementary material for: Differential effects of habitat loss on occupancy patterns of the eastern green lizard Lacerta viridis at the core and periphery of its distribution range
Source: PLoS One. 2020 Mar 5;15(3):e0229600. doi: 10.1371/journal.pone.0229600 (PMC7058328; doi:10.1371/journal.pone.0229600)
Supplement: S8 Appendix — (DOCX) [file pone.0229600.s008.docx]

S1 Appendix 8. Individual effects of non-scale and scale dependent variables.

|  |  | **Core** |  |  |  |  | **Periphery** |  |  |
| --- | --- | --- | --- | --- | --- | --- | --- | --- | --- |
|  | Estimate | Error | z | P(>\|z\|) |  | Estimate | Error | z | P(>\|z\|) |
| **Area** | 0,831 | 0,663 | 1,254 | 0,21 |  | 0,611 | 1,275 | 0,479 | 0,632 |
| **Perimeter** | -1,83 | 1,561 | -1,17 | 0,242 |  | 3,5 | 2 | 1,75 | 0,0794 |
| **Per_area** | -1,45 | 0,891 | -1,63 | 0,103 |  | 2,24 | 1,46 | 1,53 | 0,1248 |
| **Shape_index** | 3,2 | 1,789 | 1,79 | 0,073 |  | 0,094 | 0,382 | 0,246 | 0,806 |
| **Isolation** | -0,623 | 0,476 | -1,31 | 0,1903 |  | 0,152 | 0,778 | 0,196 | 0,845 |
| **Veg_str** | 15,3 | 11,6 | 1,32 | 0,185 |  | -1,45 | 8,85 | -0,1637 | 0,87 |
| **Radiation** | -0,071 | 0,452 | -0,159 | 0,873 |  | -22,5 | 26,2 | -0,857 | 0,392 |
|  |  |  |  |  |  |  |  |  |  |

| **CORE** |  |  | |  |  |  | |  |  |  |  |  |  |  |  |  |  | |  |
| --- | --- | --- | --- | --- | --- | --- | --- | --- | --- | --- | --- | --- | --- | --- | --- | --- | --- | --- | --- |
|  |  | **Habitat** | |  |  | **Crops and pastures** | |  |  |  |  | **Urban** |  |  |  |  | **Proximity index** | |  |
| Scale | Estimate | Error | z | P(>\|z\|) |  | Estimate | Error | z | P(>\|z\|) |  | Estimate | Error | z | P(>\|z\|) |  | Estimate | Error | z | P(>\|z\|) |
| 50 | 5 | 2,078 | 2,41 | 0,0161 |  | -1,88 | 1,48 | -1,27 | 0,2046 |  | -2,72 | 1,292 | -2,1 | 0,035 |  | 0,195 | 0,243 | 0,804 | 0,422 |
| 150 | 7,4 | 2,869 | 2,58 | 0,0099 |  | -1,72 | 1,395 | -1,24 | 0,2166 |  | -2,17 | 1,148 | -1,89 | 0,058 |  | 0,257 | 0,241 | 1,066 | 0,286 |
| 250 | 9,99 | 3,916 | 2,55 | 0,0107 |  | -1,83 | 1,492 | -1,22 | 0,2209 |  | -1,79 | 1,072 | -1,67 | 0,094 |  | 0,289 | 0,245 | 1,178 | 0,239 |
| 500 | 11,56 | 4,736 | 2,44 | 0,0146 |  | -1,75 | 1,64 | -1,07 | 0,285 |  | -1,642 | 1,102 | -1,49 | 0,136 |  | 0,266 | 0,244 | 1,091 | 0,275 |
| 750 | 13,43 | 6,012 | 2,23 | 0,0255 |  | -1,98 | 1,82 | -1,09 | 0,277 |  | -1,471 | 1,141 | -1,29 | 0,197 |  | 0,254 | 0,242 | 1,048 | 0,295 |
| 1000 | 14,7 | 6,584 | 2,24 | 0,0252 |  | -2,16 | 2,01 | -1,08 | 0,282 |  | -1,329 | 1,145 | -1,16 | 0,245 |  | 0,281 | 0,25 | 1,125 | 0,261 |
| 1500 | 17,84 | 9,99 | 1,79 | 0,0742 |  | -1,62 | 1,96 | -0,828 | 0,408 |  | -1,312 | 1,175 | -1,12 | 0,264 |  | 0,338 | 0,238 | 1,42 | 0,156 |
| 2000 | 26,24 | 17,26 | 1,52 | 0,129 |  | -1,05 | 1,83 | -0,572 | 0,567 |  | -1,479 | 1,28 | -1,16 | 0,247 |  | 0,349 | 0,24 | 1,455 | 0,145 |
| 2500 | 17,21 | 8,38 | 2,05 | 0,0399 |  | -0,74 | 1,79 | -0,413 | 0,679 |  | -1,588 | 1,379 | -1,15 | 0,249 |  | 0,353 | 0,241 | 1,463 | 0,144 |
| 3000 | 19,82 | 9,89 | 2 | 0,045 |  | -0,58 | 1,8 | -0,322 | 0,748 |  | -1,739 | 1,53 | -1,14 | 0,256 |  | 0,356 | 0,242 | 1,469 | 0,142 |
|  |  |  | |  |  |  |  |  |  |  |  |  |  |  |  |  |  |  |  |
| **PERIPHERY** |  |  | |  |  |  |  |  |  |  |  |  |  |  |  |  |  |  |  |
| Scale | Estimate | Error | z | P(>\|z\|) |  | Estimate | Error | z | P(>\|z\|) |  | Estimate | Error | z | P(>\|z\|) |  | Estimate | Error | z | P(>\|z\|) |
| 50 | 2 | 1,912 | 1,05 | 0,295 |  | 1,7 | 2,506 | 0,677 | 0,498 |  | -0,272 | 1,289 | -0,211 | 0,833 |  | -8,159 | 3,36E+004 | -0,0002 | 0,999 |
| 150 | 1,5 | 1,873 | 0,802 | 0,422 |  | 2,56 | 2,71 | 0,946 | 0,344 |  | -1,874 | 1,553 | -1,207 | 0,228 |  | -0,779 | 0,559 | -1,39 | 0,164 |
| 250 | 2,43 | 2,285 | 1,06 | 0,287 |  | 2,26 | 2,807 | 0,804 | 0,421 |  | -2,729 | 1,768 | -1,544 | 0,123 |  | -0,838 | 0,554 | -1,512 | 0,13 |
| 500 | 5,31 | 2,848 | 1,86 | 0,062 |  | 1,84 | 2,759 | 0,668 | 0,503 |  | -4,95 | 2,586 | -1,913 | 0,055 |  | -0,686 | 0,497 | -1,382 | 0,167 |
| 750 | 8,66 | 4,52 | 1,92 | 0,055 |  | 1,99 | 2,647 | 0,753 | 0,451 |  | -4,362 | 2,353 | -1,85 | 0,063 |  | -0,616 | 0,459 | -1,343 | 0,179 |
| 1000 | 8,37 | 4,95 | 1,69 | 0,091 |  | 1,11 | 2,661 | 0,417 | 0,676 |  | -4,712 | 2,55 | -1,849 | 0,064 |  | -0,554 | 0,434 | -1,277 | 0,202 |
| 1500 | 18,68 | 8,12 | 2,3 | 0,021 |  | -0,229 | 2,841 | -0,08 | 0,936 |  | -4,691 | 2,63 | -1,786 | 0,074 |  | -0,751 | 0,528 | -1,424 | 0,154 |
| 2000 | 23,75 | 9,68 | 2,45 | 0,014 |  | 0,99 | 3,332 | 0,297 | 0,766 |  | -4,87 | 2,74 | -1,778 | 0,075 |  | -0,832 | 0,523 | -1,592 | 0,111 |
| 2500 | 32,29 | 12,57 | 2,57 | 0,01 |  | 2,25 | 3,243 | 0,693 | 0,488 |  | -5,46 | 2,93 | -1,86 | 0,062 |  | -0,721 | 0,48 | -1,502 | 0,133 |
| 3000 | 37,25 | 15,37 | 2,42 | 0,015 |  | 3,76 | 3,39 | 1,11 | 0,267 |  | -5,75 | 3,05 | -1,89 | 0,059 |  | -0,744 | 0,502 | -1,48 | 0,139 |
|  |  |  | |  |  |  |  |  |  |  |  |  |  |  |  |  |  |  |  |
|  |  | **Dense woodland** | | |  |  | **Humid grassland** | |  |  |  |  |  |  |  |  |  |  |  |
|  | Estimate | Error | z | P(>\|z\|) |  | Estimate | Error | z | P(>\|z\|) |  |  |  |  |  |  |  |  |  |  |
| 50 | -0,053 | 2,293 | -0,023 | 0,982 |  | -20,839 | 13,853 | -1,504 | 0,133 |  |  |  |  |  |  |  |  |  |  |
| 150 | 1,22 | 2,761 | 0,44 | 0,659 |  | -14,506 | 13,76 | -1,054 | 0,292 |  |  |  |  |  |  |  |  |  |  |
| 250 | 3,39 | 3,57 | 0,949 | 0,342 |  | -14,436 | 14,572 | -0,991 | 0,322 |  |  |  |  |  |  |  |  |  |  |
| 500 | 5,55 | 3,518 | 1,58 | 0,115 |  | -24,309 | 19,676 | -1,235 | 0,217 |  |  |  |  |  |  |  |  |  |  |
| 750 | 7,09 | 3,905 | 1,82 | 0,069 |  | -37,171 | 25,54 | -1,455 | 0,146 |  |  |  |  |  |  |  |  |  |  |
| 1000 | 7,96 | 4,193 | 1,9 | 0,057 |  | -40,32 | 27,188 | -1,483 | 0,138 |  |  |  |  |  |  |  |  |  |  |
| 1500 | 8,11 | 4,293 | 1,89 | 0,059 |  | -73,026 | 37,31 | -1,957 | 0,05 |  |  |  |  |  |  |  |  |  |  |
| 2000 | 8,51 | 4,426 | 1,92 | 0,054 |  | -69,418 | 35,239 | -1,97 | 0,048 |  |  |  |  |  |  |  |  |  |  |
| 2500 | 8,5 | 4,766 | 1,78 | 0,074 |  | -73,266 | 39,725 | -1,844 | 0,065 |  |  |  |  |  |  |  |  |  |  |
| 3000 | 7,82 | 5,14 | 1,52 | 0,128 |  | -91,29 | 42,09 | -2,17 | 0,03 |  |  |  |  |  |  |  |  |  |  |
